# Improvement of quality of life, anxiety and depression after surgery in patients with stress urinary incontinence: Results of a longitudinal short-term follow-up

**DOI:** 10.1186/1477-7525-6-72

**Published:** 2008-09-29

**Authors:** Petra C Innerkofler, Verena Guenther, Peter Rehder, Martin Kopp, Dominic P Nguyen-Van-Tam, Johannes M Giesinger, Bernhard Holzner

**Affiliations:** 1 Department of Urology, Innsbruck Medical University, Anichstr.35, A-6020 Innsbruck, Austria; 2 Department of General Psychiatry, Innsbruck Medical University, Anichstr.35, A-6020 Innsbruck, Austria; 3 Centre for Occupational and Health Psychology, Cardiff University, UK; 4 Department of Biological Psychiatry, Innsbruck Medical University, Anichstr.35, A-6020 Innsbruck, Austria

## Abstract

**Objective:**

The objective of this study was to compare the effect of incontinence surgery and pelvic floor training on quality of life (QOL), anxiety and depression in patients with stress urinary incontinence (SUI).

**Methods:**

In a prospective longitudinal study, females with proven SUI were asked to complete a set of standardized questionnaires (sociodemographic data sheet, FACT-G, I-QOL, HADS) before and eight weeks after treatment. The comparison groups consisted of a surgical treatment group and a conservative group that underwent supervised pelvic floor training for eight weeks.

**Results:**

From the 67 female patients included in the study a number of 53 patients completed both assessment time points (mean age 57.4, mean years of SUI 7.6). The surgical treatment group consisted of 32 patients of which 21 patients received a modified Burch colposuspension and 11 patients a tension-free mid-urethral tape suspension. The 21 patients in the conservative group attended eight once-weekly supervised pelvic floor training sessions.

After treatment the surgical intervention group showed a significantly higher improvement of QOL (FACT-G and I-QOL) and anxiety (HADS) than the pelvic floor training group.

**Conclusion:**

For female patients with SUI surgery yielded a better outcome than pelvic floor training with regard to quality of life and anxiety.

## 1. Background

According to the International Continence Society (ICS) urinary incontinence (UI) is defined as involuntary loss of urine. It is one of the most common health problems [1-7] amongst women of nearly all ages, but there is an increasing risk in the elderly. Based on the data of their large-scale study Temml et al. [5] estimated that approximately 1 million people in Austria suffer from UI, 850 000 of these are women.

There are different types of UI including stress urinary incontinence (SUI), urge incontinence, mixed incontinence, neurogenic incontinence, functional incontinence or overflow incontinence [8]. This study is restricted to patients with SUI.

The majority of patients suffering from UI have weakened pelvic floor muscles. In the case of SUI an increase in intraabdominal pressure (induced by activities such as coughing, laughing, sneezing, lifting of heavy loads or using stairs) causes involuntary urinary leakage without contraction of the bladder muscles [2,9,10]. SUI often occurs when this is combined with a change of position of the bladder with increasing intraabdominal pressure such that the muscles that force the urethra to shut are prevented from squeezing as tightly as they should. As a result, urine may leak during moments of physical exercise. SUI also occurs if the sphincter muscles weaken, e.g. after childbirth, after pelvic floor muscles disorders or following anatomical changes such as after pelvic operations. Because of this dislocation of bladder and bladder neck the increasing abdominal pressure cannot be transmitted to the urethra and it therefore bears on the bladder giving rise to involuntary leakage of urine [11,12]. In addition the lack of oestrogen after menopause also plays an accessory role by causing morphological and functional change in the urogenital tract of women [13].

Many studies show that urinary incontinence has a negative impact on the lifestyle of the patients and affects emotional, social, physical and sexual aspects of well-being [3,6]. Incontinent women often avoid social contact because of feelings of shame, which negatively influences their quality of life (QOL). Incontinence sufferers may also experience anxiety arising from concerns about whether they will reach the toilet in time. This may lead patients to abstain from all sorts of social activities, such as visiting friends, sport, shopping or going to work [6]. Furthermore an association has been found between higher levels of anxiety [14,15] and depressive symptoms in women with urinary incontinence [16-20]. Nygaard et al. [19] showed in their study, that patients who suffer from UI have a smaller social network and take part in fewer public activities, which could contribute to the development of depressive symptoms. Fultz and Herzog [17] found that the involuntary loss of urine leads to despair and inferiority feelings. Furthermore patients have reported lack of self-confidence and being left alone with their problems as well as shame and loss of vitality [4].

The treatment of SUI should start when it becomes a cause of concern to the patient. Before surgical treatment is considered, conservative treatment, such as pelvic floor training is recommended [21]. Studies show that pelvic floor muscle exercises with biofeedback and electrical stimulation are an effective treatment of female SUI, even in the long term [22]. However, it has also been shown, that a high percentage (31 – 47%) underwent incontinence surgery during the following year because of persistent symptoms [23]. If pelvic floor training is not successful, incontinence surgery such as the modified Burch colposuspension, retropubic tension-free vaginal tape (TVT^®^) or transobturator urethral tape suspension can be considered [21]. The Burch colposuspension involves fastening the lateral vaginal wall to Coopers' ligament in a tension free fashion. The suspended anterior vaginal wall functions as a hammock [24]. Mid-urethral tape suspension with the new techniques (TVT^®^, SPARC^®^; MONARC^®^) fixes the urethra, especially in moments of increased intra-abdominal pressure [25].

The literature shows, that, in spite of the high prevalence and negative consequences of UI, only a low percentage of women seek treatment [4,5,26-28]. In this context many researchers emphasize that many patients have a lack of information concerning incontinence and its treatment options [5,26].

Up to present only a few studies have investigated QOL after incontinence surgery. These have generally shown that the symptoms in most patients were reduced after treatment which lead to an increase of different aspects of well-being [29-31]. Kulseng-Hanssen et al. [29] showed, that 5 to 10 years after a Burch colposuspension 75% of the patients were continent during the 24 h-pad-test and a stress test and that concern increased with symptom intensity. Most previous studies used quantity of leaking urine as an objective clinical outcome, whereas this study focuses on important psychosocial variables.

## 2. Methods

### 2.1 Purpose of the study

The main objective of this longitudinal study was to evaluate the impact of surgery and pelvic floor training on anxiety, depression and various aspects of QOL.

The following questions and hypotheses were addressed in detail:

1.) Is surgical treatment superior to pelvic floor training in patients with clinically proven SUI with regard to QOL?

Hypothesis: Patients undergoing surgery show higher improvement in QOL-scores at 8-week-follow-up with regard to the FACT-G and I-QOL than patients with pelvic floor training.

2.) Is surgical treatment superior to pelvic floor training in patients with clinically proven SUI with regard to anxiety and depression?

Hypothesis: Patients undergoing surgery show higher improvement in anxiety- and depression-scores at 8-week-follow-up with regard to the HADS than patients with pelvic floor training.

3.) Are disease-specific QOL- instruments more sensitive towards improvement or deterioration over time than generic ones?

Hypothesis: Effect sizes for changes of QOL-scores over time are larger for the disease-specific I-QOL than for the generic FACT-G.

### 2.2 Sample

In the presented non-randomized study, female patients with diagnosed SUI attending the outpatient unit of the Departments of Urology and Gynecology at Innsbruck Medical University and the Department of Urology at Hall County Hospital were consecutively included over a period of one year. The inclusion criteria were: informed consent, clinical diagnosis of SUI, age over 18 years and fluency in German. Exclusion criteria were the presence of urological or gynecological cancer and cognitive impairments.

The patients were allocated to the surgical group, if they underwent surgical treatment like the modified Burch colposuspension (i.e. lateral tension-free vaginal suspension), tension-free-vaginal-tape (TVT), SPARC or MONARC. The conservative group included patients that were on the so-called "waiting list" for surgery. They took part in eight once-weekly training sessions to strenghten their pelvic floor muscles. Supervision was done by a specialized pelvic floor physiotherapist. Treatment included an initial bimanual pelvic floor muscle evaluation, individual biofeedback training and group sessions at least one hour at a time.

All patients received instructions for pelvic floor muscle training on their first consultation for UI. For at least 6 weeks all patients tried pelvic floor muscle training at home, before a decision for surgery was made.

**Figure 1:**
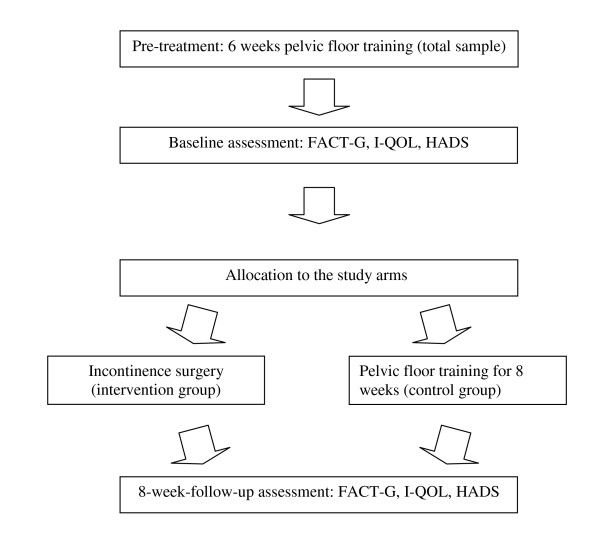
Flow-chart for study design.

The study used a longitudinal design comprising two assessment time points. The patients were asked to answer a set of questionnaires before treatment and then 8 weeks after surgery or after completion of eight once-weekly pelvic floor training sessions. The study design is detailed in the flow chart presented in Figure 1.

All subjects filled in a data sheet which recorded sociodemographic data, a generic QOL questionnaire, the Functional Assessment of Cancer Therapy Scale – General (FACT-G), a stress incontinence specific assessment instrument, the Incontinence Quality of Life Instrument (I-QOL) and the Hospital Anxiety and Depression Scale (HADS). Clinical data were recorded from the medical charts.

### 2.3 Assessment instruments

#### Functional Assessment of Cancer Therapy Scale – General (FACT-G)

The FACT-G (version 4) is the core questionnaire of a collection of QOL inventories focusing on chronic illnesses [32]. It is used internationally and has undergone extensive psychometric testing. The FACT-G is designed for self-assessment and consists of 27 items to be rated on a five-point-Likert scale. Each question of the inventory is scored from 0 (worst possible QOL) to 4 (best possible QOL). In addition to an overall QOL score (the sum of all items), there are subscales for the domains of physical well-being, social well-being, emotional well-being and functional well-being.

#### Incontinence Quality of Life Instrument (I-QOL)

The Incontinence Quality of Life Instrument (I-QOL) [33] is a self-report QOL measure for evaluating the perceived impact of UI on health-related QOL. The 22 items of the I-QOL are answered on a five-point-Likert scale. Example items are "I worry about not being able to get to the toilet on time", "I worry about coughing and sneezing" and "I have to be careful about standing up after sitting down".

Item scores are summed to produce an overall total score. A higher score represents better QOL. The I-QOL contains 3 subscales: Avoidance and limiting behaviour (8 items), Psychosocial impacts (9 items) and Social embarrassment (5 items). The overall I-QOL summary score showed high internal consistency (Cronbach's alpha 0.95) and high retest-reliability (0.91). Each subscale also showed acceptable alpha values (0.87–0.93) [33].

#### Hospital Anxiety and Depression Scale (HADS)

The Hospital Anxiety and Depression Scale (HADS) [34] is a widely used self-rating scale for detecting anxiety and depression and has been shown to have good psychometric properties [35,36]. The anxiety (HADS-A) and depression (HADS-D) subscales consist each of 7 items (scores ranging 0–21). The subscales have also been shown to be valid measures of severity of emotional disorders in clinical populations with physical comorbidities [37].

### 2.4 Statistical methods

Subscores of the questionnaires (FACT-G, I-QOL, HADS) were calculated according to the instructions of the developers [32-34].

A general linear model (GLM) for repeated measures was used to investigate the long-term effect of surgical treatment and pelvic floor training on anxiety, depression and the assessed aspects of quality of life. Assessment time points (before and 8 weeks after treatment) were included as within-subject factor and treatment (surgical treatment vs. pelvic floor training) as between-subject factor. The various employed scales were used as dependent variables each at a time. Thus the model was capable of testing differences of the impact of treatment, overall changes in time and treatment-independent group differences. To determine effect sizes partial Eta squared (ή_p_^2^) were calculated. Partial Eta squared specifies what proportion of the sum of error variance and a certain effect variance is explained by this effect in the sample: ή_p_^2 ^= SS_effect_/(SS_effect _+ SS_error_)

Additionally T-tests for dependent and independent samples were used.

For comparisons regarding sociodemographic and clinical variables Pearson-χ^2^-tests, Mann-Whitney-U-tests and T-tests were conducted.

## 4. Results

### 4.1 Patient characteristics

Within the study time frame of one year a number of 67 patients met inclusion criteria and were therefore eligible for participation in the study. All of them (100%) signed informed consent.

Of these 33 patients (49.3%) were allocated to the surgical treatment group and 34 patients were assigned to the pelvic floor training group.

One patient from the surgical treatment group and 13 patients from the pelvic floor training group dropped out of the study before the second assessment time point. The latter patients gave lack of time as the main reason for failing to complete the training sessions. No significant differences with regard to sociodemographic and clinical variables as well as all assessment instruments used were found between patients who dropped out and those who finished the study. Thus, 53 patients were available for statistical analyses.

In the surgical treatment group 21 patients (65.6%) were treated with the modified Burch colposuspension and 11 patients (34.4%) with a tape suspension. The mean age for the whole sample was 57.4 years (SD 9.4) and average time since initial diagnosis was 7.6 years (SD 8.2)

A number of 10 patients (18.9%) had previous UI surgery and 25 patients (47.2%) had a hysterectomy. The average number of child births per patient was 2.3 (SD 1.1).

Besides menopausal status (χ^2 ^= 10.40, p = 0.01) there were no statistically significant differences between the surgical treatment group and the pelvic floor training group regarding the assessed sociodemographic and clinical data. Differences in age and the frequency of episiotomy just failed significiance (each p = 0.06). For a detailed description of the sociodemographic and clinical data see Table 1.

**Table 1:** Sociodemographic and clinical data

		**Surgery ** (N = 32)	**Pelvic floor training ** (N = 21)	**Total ** (N = 53)	
**Age**	mean (SD)	59.8 (9.2)	54.5 (9.0)	57.4 (9.4)	t = 1.96, p = 0.06
	range	37 – 78	38 – 68	37 – 78	

**Marital status**	unmarried/single	9.4%	0.0%	5.7%	χ^2 ^= 3.69, p = 0.30
	married/partnership	78.1%	90.5%	83.0%	
	divorced/separated	6.3%	0.0%	3.8%	
	widowed	6.3%	9.5%	7.5%	

**Education**	primary school	40.6%	33.3%	37.7%	χ^2 ^= 1.74, p = 0.63
	completed apprenticeship	46.9%	61.9%	52.8%	
	A-level/university	12.5%	4.8%	9.4%	

**Occupational status**	full time work	9.4%	9.5%	9.4%	χ^2 ^= 7.18, p = 0.07
	part time work	15.6%	38.1%	24.5%	
	housewife	15.6%	28.6%	20.8%	
	retired	59.4%	23.8%	45.3%	

**Years of urinary stress incontinence**	mean (SD)	7.2 (7.8)	8.3 (8.8)	7.6 (8.2)	Z = -.0.63, p = 0.53
	range	0.2 – 31.0	2.0 – 38.0	0.2 – 38.0	

**Number of births**	mean (SD)	2.3 (1.2)	2.2 (1.1)	2.3 (1.1)	Z = -0.21, p = 0.83
	0	3.1%	0.0%	1.9%	
	1	18.8%	19.0%	18.9%	
	2	46.9%	52.4%	49.1%	
	≥ 3	31.2%	28.6%	30.1%	

**Episiotomy**	yes	40.6%	66.7%	50.9%	χ^2 ^= 3.44, p = 0.06
	no	59.4%	33.3%	49.1%	

**Caesarian section**	yes	6.3%	0.0%	3.8%	χ^2 ^= 1.36, p = 0.24
	no	93.8%	100.0%	96.2%	

**Birth weight of the heaviest child**	mean (SD)	3485 (468)	3620 (494)	3540 (479)	t = -0.99, p = 0.33
	< 3000 g	10.0%	9.5%	9.8%	
	3000 – 3999	73.3%	61.9%	68.6%	
	≥ 4000	16.7%	28.6%	21.6%	

**Body Mass Index**	mean (SD)	26.6 (4.2)	27.1 (5.9)	26.8 (5.0)	Z = -0.19, p = 0.85
	range	19.5–39.4	20.8–47.0	19.5–47.0	

**Menopausal status**	before menopause	9.4%	28.6%	17.0%	χ^2 ^= 10.40, p = 0.01
	in menopause	21.9%	47.6%	32.1%	
	after menopause	68.8%	23.8%	50.9%	

**Psycholog./psychiatric treatment**	yes	15.6%	9.5%	13.2%	χ^2 ^= 0.41, p = 0.52
	no	84.4%	90.5%	86.8%	

### 4.2 Anxiety and depression in patients undergoing surgical treatment or pelvic floor training

There were no statistically significant differences in anxiety and depression measured with HADS between the surgical treatment group and the pelvic floor training group at baseline. In the pelvic floor trainnig group differences in anxiety and depression between baseline and 8-weeks-follow-up were not signficant, whereas both scales differed significantly between the two assessment time points in the surgical treatment group.

**Figure 2:**
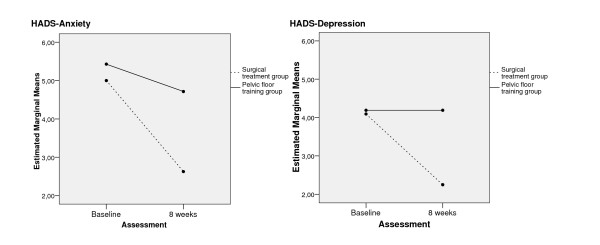
Changes in anxiety and depression (HADS).

The change in depression over time did not differ significantly between the two groups. The Anxiety-scale however showed a significantly stronger decrease in the surgical treatment group than in the pelvic floor training goup (see Table 2, Table 3 and Figure 2).

**Table 2:** Descriptive statistics for anxiety and depression (HADS*) and quality of life (FACT- G** and I-QOL**)

	Baseline			8-weeks		
	Surgical treatment	Pelvic floor training		Surgical treatment	Pelvic floor training	
	Mean (SD)	Mean (SD)		Mean (SD)	Mean (SD)	
HADS						
Anxiety	5.0 (3.5)	5.4 (2.7)	t = -0.50; p = 0.619	2.6 (2.4)	4.7 (3.1)	t = -2.74; p = 0.008
Depression	4.1 (3.6)	4.2 (2.7)	t = -0.11; p = 0.917	2.3 (3.5)	4.2 (3.2)	t = -2.00; p = 0.046

FACT-G						
Physical well-being	23.0 (5.9)	24.9 (3.8)	t = -1.31; p = 0.196	25.2 (3.7)	25.4 (2.6)	t = -0.28; p = 0.784
Emotional well-being	19.2 (3.6)	18.2 (3.7)	t = 0.92; p = 0.363	21.6 (2.8)	18.3 (3.3)	t = 3.88; p < 0.001
Functional well-being	20.3 (6.0)	20.3 (3.7)	t = 0.04; p = 0.965	23.0 (4.7)	20.5 (3.7)	t = 2.05; p = 0.046
Social well-being	16.7 (5.4)	12.7 (3.5)	t = 3.21; p = 0.002	18.3 (4.3)	14.5 (5.5)	t = 2.82; p = 0.007
Total	79.2 (15.6)	76.0 (9.2)	t = 0.92; p = 0.360	88.1 (12.1)	78.7 (10.1)	t = 2.89; p = 0.006

I-QOL						
Avoidance	24.8 (7.6)	26.1 (6.7)	t = -0.66; p = 0.511	36.5 (4.6)	28.7 (6.5)	t = 4.74; p < 0.001
Psychosocial impact	33.9 (7.8)	37.4 (5.7)	t = -1.78; p = 0.081	42.9 (3.1)	38.7 (6.2)	t = 2.89; p = 0.008
Social embarrassment	13.8 (5.9)	16.7 (4.9)	t = -1.84; p = 0.072	23.7 (2.5)	18.3 (4.6)	t = 4.89; p < 0.001
Total	72.5 (18.2)	80.2 (15.7)	t = -1.59; p = 0.117	103.1 (9.2)	85.7 (16.5)	t = 4.41; p < 0.001

* higher scores indicates more anxiety and depression

** higher value indicates better quality of life

**Table 3:** Group, time and interaction effects from the general linear model for repeated measures for anxiety and depression (HADS) and quality of life (FACT- G and I-QOL)

	Group			Time			Group-time-interaction		
	F	p	ή_p_^2^	F	p	ή_p_^2^	F	p	ή_p_^2^
HADS									
Anxiety	2.84	0.098	0.053	16.78	< 0.001	0.248	4.85	0.032	0.087
Depression	1.64	0.206	0.031	3.47	0.068	0.064	3.47	0.068	0.064

FACT-G									
Physical Well-being	0.90	0.346	0.017	9.372	0.004	0.155	3.32	0.074	0.061
Emotional Well-being	7.50	0.008	0.128	5.53	0.023	0.098	4.73	0.034	0.085
Functional Well-being	1.02	0.318	0.020	9.45	0.003	0.157	7.09	0.010	0.122
Social Well-being	10.17	0.002	0.169	8.34	0.006	0.143	0.01	0.913	> 0.001
Total	3.55	0.065	0.066	21.12	< 0.001	0.297	5.99	0.018	0.107

I-QOL									
Avoidance	4.68	0.035	0.084	50.82	< 0.001	0.499	50.52	< 0.001	0.287
Psychosocial Impact	0.06	0.805	0.001	27.76	< 0.001	0.352	15.62	< 0.001	0.234
Social Embarrassment	1.47	0.23	0.028	56.79	< 0.001	0.527	29.30	< 0.001	0.365
Total	1.93	0.171	0.036	56.07	< 0.001	0.524	27.03	< 0.001	0.346

Adding menopausal status as a between-subject factor to the GLM also did not affect the results for anxiety and depression (not shown).

### 4.3 QOL in patients undergoing surgical treatment or pelvic floor training

At baseline no statistically significant differences regarding QOL were found between the surgical treatment group and the pelvic floor training group with the exception of FACT-G Social Well-being (surgical treatment: mean = 16.7 vs. pelvic floor training: mean = 12.7; p = 0.002).

**Figure 3:**
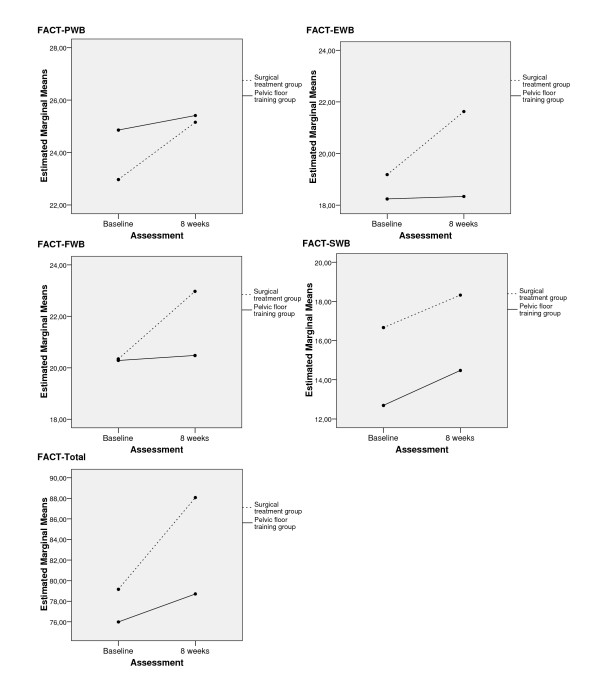
Changes in physical well-being (FACT-PWB), emotional well-being (FACT-EWB), functional well-being (FACT-FWB), social well-being (FACT-SWB) and FACT-Total.

Increase in QOL between baseline and 8-weeks-follow-up reached significance (p < 0.05) for all FACT-G and I-QOL scales in the surgical treatment group. In the pelvic floor training group only the I-QOL-scale Avoidance had a significant increase (p = 0.045). The GLM for repeated measures showed that the FACT-G scales Emotional Well-being, Functional Well-being and Total-Score showed a significantly higher improvement over time in the surgical treatment group than in the pelvic floor training group (see Table 2, Table 3 and Figure 3).

**Figure 4:**
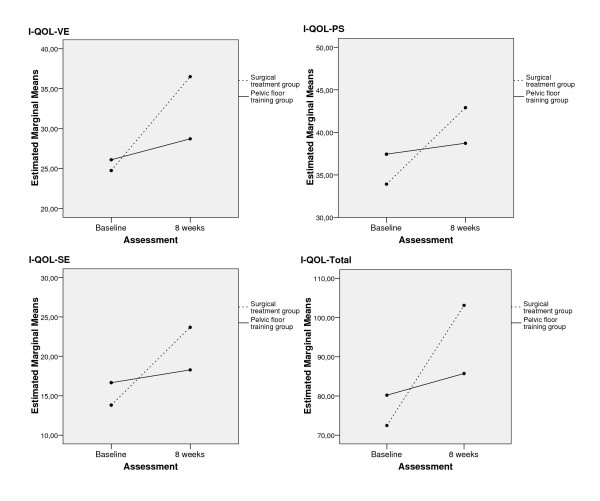
Changes in avoidance and limiting behavior (I-QOL-VE), psychosocial impact (I-QOL-PS), social embarrassment (I-QOL-SE) and I-QOL-Total.

Furthermore, there were significantly higher increases in the surgical treatment group regarding the I-QOL-scales Avoidance, Psychosocial-Impact, Social Embarrassment and Total-Score (see Table 2, Table 3 and Figure 4).

Adding menopausal status as a between-subject factor to the GLM did not affect the results for QOL (not shown).

The intervention effect sizes were about four times bigger for the aspects of QOL that were covered by the I-QOL than for those assessed by the FACT-G (average ή_p_^2 ^= 0.308 vs. 0.075).

## 5. Discussion

It is well known that UI influences emotional, social and physical aspects of well-being and has a negative impact on the patients' QOL [3,6].

Surgical treatment is considered when conservative treatment (in particular pelvic floor training) is ineffective. Studies regarding this topic have primarily focused on clinical parameters such as leaking of urine. In contrast psychological outcome variables (i.e. psychological well-being) have usually been regarded as being only of secondary importance.

The aim of this longitudinal study was to compare surgical treatment and pelvic floor training in patients with clinically proven SUI with regard to QOL, anxiety and depression. The main focus was the effect of surgery and pelvic floor training on the course of patients' subjective well-being.

At baseline (apart from social well-being) no relevant differences were found for QOL anxiety and depression between the surgery group and the pelvic floor training group.

The FACT-G Social Well-being scale comprises mainly items regarding social support rather than participation in social acitivities. The finding that at baseline patients in the surgical treatment group had higher scores on the dimension of social well-being may be because, as Swithinbank et al. [6] and Berglund et al. [7] have suggested, incontinence is a taboo subject and incontinent women have difficulty talking about it, especially to their husbands. It can be assumed that because surgery necessitates a hospitalisation, a lot of patients were forced to inform their families about their disease. This may have increased acceptance of the disease and received social support.

As expected the general FACT-G scales were less sensitive towards changes in QOL over time than the disease-specific I-QOL scales. This resulted in considerably smaller effect sizes for the FACT-G scales, i.e. a smaller proportion of explained variance compared with error variance.

Since the FACT-G Physical Well-being scale covers a more severe range of physical impairment than usually found in patients with SUI, a strong ceiling effect occured and no significant difference between the two treatment groups was shown.

For Emotional Well-being, Functional Well-being and global QOL however the surgical treatment group showed a significantly higher improvement over time than the pelvic floor training group.

Regarding the I-QOL the surgical treatment group yielded a significantly better outcome for all scales (Avoidance and Limiting Bevahior, Psychosocial Impact, Social Embarrassment and I-QOL-Total).

All patients had relevant problems regarding Psychosocial Impact before treatment such that, one way or the other, all patients became socially withdrawn and for example avoided having to meet in public places or at public events. These results are acknowledged by Swithinbank et al. [6]. According to their study incontinence may cause withdrawal from social activities and the women with incontinence have less social interaction than women without involuntary loss of urine. Similar results from Berglund et al. [7] describe diminished social integration and loneliness as a result of it. This may contribute to the development of anxiety or depressive symptoms and reduce global QOL.

The results with regard to the dimension of Social Embarrassment were similar to those of Psychosocial Impact. Before treatment many women described their experience with incontinence as embarrassing and so often stayed at home because they were anxious about not having ready access to a toilet and were fearful of an urinary accident in public. In this context Bogner et al. [38] describe the loss of self-confidence caused by the feeling of shame. This can also cause a further withdrawal from social activities.

After incontinence surgery patients also had a significantly better outcome for Avoidance and Limiting Behaviour than the conservative group. Before surgical treatment patients often worried, if they did not know where the toilets were, that they had to plan every detail in advance because of their UI and that they were afraid of physical activities, because of the association between involuntary loss of urine and physical strain. The outcome of these limitations is once more social retreat and in worst case social isolation of the patient (according to Bogner et al. [38] this limiting of social and physical activities is "condition-specific functional loss"). This may be self-imposed by the patients to help managing their condition, but it may lead to feelings of loss of control and distress and it diminishes life satisfaction.

Thus the results from the I-QOL scales and the FACT-G scales are concordant since decreased social withdrawal and avoidance, reduced psychosocial impact and less embarrassment are accompanied by better emotional and functional well-being.

Not assessed in our study but nevertheless of importance is the negative impact of SUI on women's sexual functioning and sexual well-being. As Oh et al. [39] pointed out a relevant proportion of women suffering from SUI reports pain during intercourse and coital incontinence, that have a detrimental effect on overall well-being.

Another aim of the study was to determine whether the effect of surgical treatment or pelvic floor training differs regarding anxiety and depression. Improvement regarding anxiety was significantly higher in the surgical treatment group than in the pelvic floor training group, whereas differences in changes for depression failed significance.

To compare anxiety and depression in this sample of women suffering from SUI with age-matched women in the general population we used reference data from Hinz and Schwarz [40]. Their study provides norm values for the HADS from a representative sample of the German adult population (n = 2037). For women aged 40–59 they report for the HADS anxiety scale a mean value of 5.2 (SD 3.4) and for depression a mean value of 4.8 (SD 3.7). These scores are very similar to those found for the pelvic floor training group at baseline and after treatment and for the surgical treatment group at baseline. Eight weeks after treatment however the surgical treatment group showed notably lower average scores regarding anxiety (2.6) and depression (2.3).

The findings of our study concerning depression are somewhat different to other studies, e.g. Steers et al. [16] and Fultz et al. [17] reported heightened values of depression in patients with urinary incontinence. Depression would also seem to be consistent with negative emotions and feeling of inferiority caused by involuntary loss of urine.

Some limitations of the study should be mentioned. The exact degree of SUI (i.e. pad test) was not investigated. Thus, it cannot be evaluated, if a higher degree of UI causes a higher degree of psychological burden to the patients. The main focus of treatment, however, was the subjective feelings of the patient and for this reason questionnaires as self-rating instruments were used. Furthermore, it is noted that to achieve maximum efficacy, pelvic floor training requires daily training in addition to the once-weekly training sessions. The extent of patient compliance with this additional daily training is unknown. Lack of compliance with the once-weekly training can be seen as the reason for not completing the 8-weekly pelvic floor training sessions in 13 patients. On the other hand the problem of limited compliance not only occurs in this study sample but is a general drawback of pelvic floor training.

A further limitation is that the follow-up-assessment took place only eight weeks post-operatively. In view of the fact that the healing process after surgery takes months, it can be expected, that the QOL of the patients will even improve further. Nevertheless it has to be pointed out, that the long-term effect of surgery is again dependent on the strength and functional activity of the pelvic floor muscles.

Finally, comparability of the surgical treatment group and the pelvic floor training group might be affected by unknown confounders since for ethical reasons randomization was not possible.

In spite of the favourable outcome of the surgical treatment procedure in this study, we are aware of the fact, that it should never be forgotten that surgery always contains risks for the patients. According to Broome [21] pelvic floor training could be considered as part of conservative first-line therapy. Taking into account the natural progression of the disease appropriately scheduled follow-up examinations may be the basis for initiating surgery.

## 6. Conclusion

In summary, for SUI patients eight weeks after treatment, surgery (modified Burch colposuspension, tension-free mid-urethral tape suspension) yielded a better outcome with regard to QOL and anxiety than pelvic floor training. Longterm follow-up is planned to determine whether this difference is still present one year after treatment.

## Competing interests

The authors declare that they have no competing interests.

## Authors' contributions

IP and GV were responsible for study design and conceptualization as well as for data collection. RP was one of the treating urologists and therefore in charge of patient recruitment. GJ and KM performed the statistical analysis. HB and ND helped to draft the manuscript and gave important input for intellecutal content.
